# A short history from Karelia study to biodiversity and public health interventions

**DOI:** 10.3389/falgy.2023.1152927

**Published:** 2023-03-14

**Authors:** Tari Haahtela, Harri Alenius, Petri Auvinen, Nanna Fyhrquist, Leena von Hertzen, Pekka Jousilahti, Piia Karisola, Tiina Laatikainen, Jenni Lehtimäki, Laura Paalanen, Lasse Ruokolainen, Kimmo Saarinen, Erkka Valovirta, Tuula Vasankari, Tiina Vlasoff, Marina Erhola, Jean Bousquet, Erkki Vartiainen, Mika J. Mäkelä

**Affiliations:** ^1^ Skin and Allergy Hospital, Helsinki University Hospital, Helsinki, Finland; ^2^ University of Helsinki, Helsinki, Finland; ^3^ Human Microbiome Research (HUMI), Faculty of Medicine, University of Helsinki, Helsinki, Finland; ^4^ Institute of Environmental Medicine, Karolinska Institutet, Stockholm, Sweden; ^5^ DNA Sequencing and Genomics Laboratory, Institute of Biotechnology, Helsinki, Finland; ^6^ Department of Public Health and Welfare, Finnish Institute for Health and Welfare (THL), Helsinki, Finland; ^7^ Institute of Public Health and Clinical Nutrition, University of Eastern Finland, Kuopio, Finland; ^8^ Finnish Environment Institute, Helsinki, Finland; ^9^ Department of Biosciences, University of Helsinki, Helsinki, Finland; ^10^ University of Eastern Finland, Joensuu, Finland; ^11^ Department of Pulmonary Diseases and Clinical Allergology, University of Turku, Turku, Finland; ^12^ Allergy Clinic, Terveystalo, Turku, Finland; ^13^ Finnish Lung Health Association (FILHA), Helsinki, Finland; ^14^ North Karelia Centre for Public Health, Joensuu, Finland; ^15^ Pirkanmaa Joint Authority for Health Services and Social Welfare, Tampere, Finland; ^16^ Institute of Allergology, Charité — Universitätsmedizin Berlin, Corporate Member of Freie Universität Berlin and Humboldt-Universität zu Berlin, Berlin, Germany; ^17^ Institute for Translational Medicine and Pharmacology ITMP, Allergology and Immunology, Berlin, Germany; ^18^ University Hospital Montpellier, Montpellier, France

**Keywords:** allergy epidemic, allergy programme, asthma, biodiversity hypothesis, climate change, Karelia Allergy Study, nature loss, non-communicable diseases

## Abstract

Contact with natural environments enriches the human microbiome, promotes immune balance and protects against allergies and inflammatory disorders. In Finland, the allergy & asthma epidemic became slowly visible in mid 1960s. After the World War II, Karelia was split into Finnish and Soviet Union (now Russia) territories. This led to more marked environmental and lifestyle changes in the Finnish compared with Russian Karelia. *The Karelia Allergy Study 2002–2022* showed that allergic conditions were much more common on the Finnish side. The Russians had richer gene-microbe network and interaction than the Finns, which associated with better balanced immune regulatory circuits and lower allergy prevalence. In the Finnish adolescents, a biodiverse natural environment around the homes associated with lower occurrence of allergies. Overall, the plausible explanation of the allergy disparity was the prominent change in environment and lifestyle in the Finnish Karelia from 1940s to 1980s. The nationwide *Finnish Allergy Programme 2008–2018* implemented the biodiversity hypothesis into practice by endorsing immune tolerance, nature contacts, and allergy health with favorable results. A regional health and environment programme, *Nature Step to Health 2022–2032*, has been initiated in the City of Lahti, EU Green Capital 2021. The programme integrates prevention of chronic diseases (asthma, diabetes, obesity, depression), nature loss, and climate crisis in the spirit of *Planetary Health*. Allergic diseases exemplify inappropriate immunological responses to natural environment. Successful management of the epidemics of allergy and other non-communicable diseases may pave the way to improve human and environmental health.

## 1. Background

The *biodiversity hypothesis* of allergy, and health in general, was presented in 2011–2012 (1, 2). It was acknowledged by the World Allergy Organization as a position statement in 2013 (3), highlighted in 2013 (4), and reviewed in 2019 (5, 6). It has gained attention as it combines human health with environmental and lifestyle determinants as well as medical discipline with ecology. The recent concept of *Planetary health* has further emphasized the dependence of human civilization on the state of natural systems and planetary boundaries (7, 8).

Biodiversity was defined in 1992 by United Nations as the variability among living organisms from all sources, including inter alia, terrestrial, marine and other aquatic ecosystems and the ecological complexes of which they are part (9). This includes diversity within species, between species and ecosystems. In practice, increasing ecosystem diversity promotes stability through functional redundancy, broader utilization of available resources, weak among-species interactions, and alternative energy channels (10).

Here, we outline the short history of the Karelia Allergy Study leading to the biodiversity hypothesis and to practical actions to mitigate burden of asthma & allergy and endorse human health. The hypothesis also opens up a rationale to integrate public health promotion to measures combating the major challenges of our time; climate crisis and nature loss.

## 2. From tuberculosis to asthma and allergic disease

In 2002, Jean-Francois Bach reported on the inverse relation between the incidence of infectious diseases like tuberculosis, and immune disorders like asthma, from 1950s to 2000s (11). In 1970s, the transition was also experienced in Finland. Along with the new and effective medication, tuberculosis patients “started to march out of the old sanatoria and asthma patients in”.

While asthma and allergy patients increased their treatment was inadequate. Before the introduction of inhaled corticosteroids, asthma medication, mainly inhaled short-acting *β*2-agonists like salbutamol and oral theophylline, was not helping the patients in any longer term, and oral corticosteroids were not safe. The patients kept on coughing, producing mucus and wheezing. Beclomethasone dipropionate was the first inhaled corticosteroid (ICS), introduced in 1972, but was not used for mild or early asthma.

**Figure 1:**
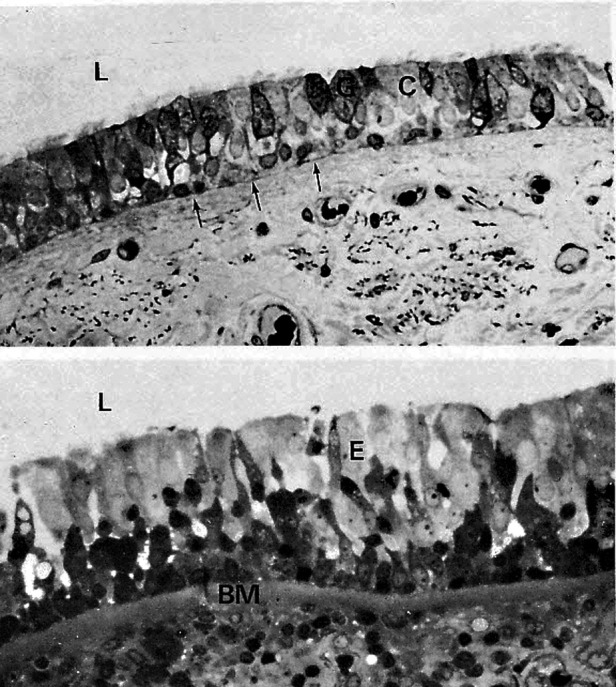
Upper light microscopical picture shows normal bronchial epithelium (L = lumen, C = ciliated cells, G = goblet cells). Thin arrows show the basement membrane (BM). Lower picture shows bronchial epithelium (**E**) from a patient with newly detected asthma. There Is edema fluid between epithelial cells, BM Is swollen, and the tissue under BM (lamina propria) is full of eosinophils (black dots) (12, modified).

In 1985, Lauri A. Laitinen and coworkers showed that even patients with newly detected asthma had damage of the bronchial wall, i.e., mucosal swelling, opening of the tight junctions between the ciliated cells, and inflammatory cell influx (Figure 1) (12). In 1990, Jean Bousquet and coworkers confirmed that eosinophils play a central role in the asthmatic inflammation (13). Cezmi A. Akdis and his group made innovative studies on the mechanisms of the epithelial processes and leakage introducing in 2021 the *epithelial barrier hypothesis* of allergy and autoimmunity (14).

In genetically susceptible individuals, the primary inflammation may be followed by increased bronchial responsiveness, remodeling of the bronchial wall, persistent airflow limitation and chronic disease (15). A nationwide 10-year asthma programme was implemented in Finland between 1994 and 2004 to improve early detection and treatment of inflammation. The change of practice was based on a controlled, long-term intervention showing superiority of ICS over *β*2-agonist as a first-line medication (16). A major change for the better was achieved (17, 18) and adopted in other regional and national activities in Europe and elsewhere (19, 20).

### 2.1. Epidemic of asthma and allergy

**Figure 2:**
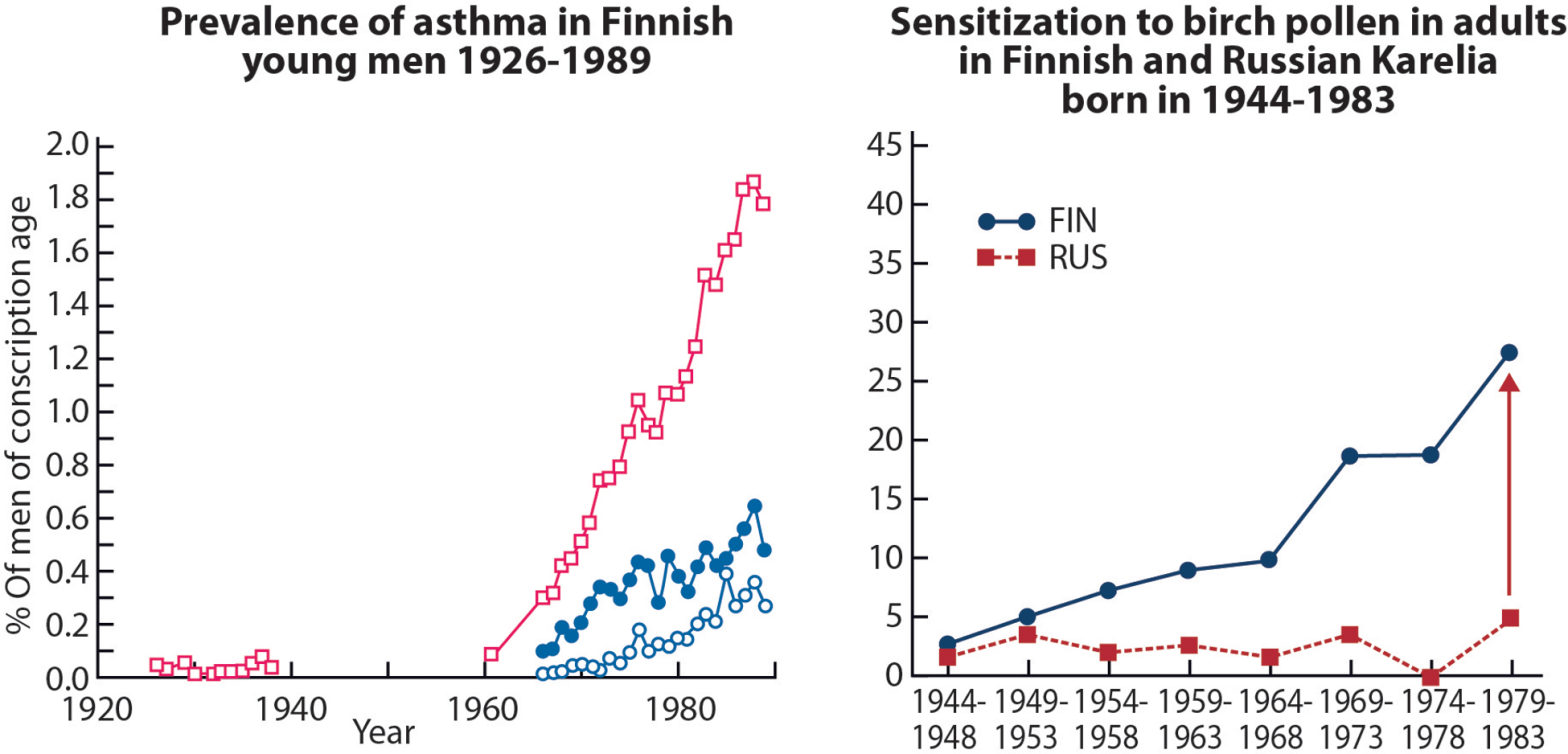
Left panel, asthma prevalence in the Finnish conscripts 1926–1989 (21). Two lower curves indicate percentages of men exempted at call-up medical examination because of asthma (closed circles), and those discharged during course of the service (open circles). Right panel, generational increase in positive allergen specific IgE levels to birch pollen in the Finnish but not in the Russian Karelia (22). Among older generations, born in the 1940s, the prevalence was at the same low level in Finnish vs. Russian Karelia. Adopted from Ref. (5).

The main question remained, however. What is causing the inflammation in the first place? Why did the population seem ever more prone to airway inflammatory responses? In the Finnish young men, military conscripts, the rise of asthma and allergic rhinitis became apparent in the 1960s (21) (Figure 2, left panel). Many were allergic to pollen, house dust mite, animal danders, and small children were increasingly allergic to various food items. But also subjects without any signs of IgE-sensitization, i.e., atopic constitution, showed asthma and rhinitis, often provoked by respiratory viral infections (23).

## 3. Allergy disparity in the Finnish and Russian Karelia

In 1972, a large community-based intervention was initiated to decrease the high cardiovascular disease mortality in the province of North Karelia, Eastern Finland, with highly successful results (24). The FINRISK study has expanded the intervention to major non-communicable diseases and the entire nation since 1976 (25).

In the late 1990s, Erkki Vartiainen suggested a FinAllergy study. An opportunity opened up not only to study asthma and allergy in Finland but also to compare Finnish and Russian Karelia (26). These adjacent areas had been strictly separated by the *iron curtain* after the World War II. Urbanization, industrialization and an improved living standard took place on the Finnish side (westernization) while the population on the Russian side continued their small-scale, self-supporting agricultural lifestyle. Soviet leaders left parts of Russian Karelia near the border as kind of a barrier against western influence. A living laboratory was born to study impact of lifestyle, environment, microbiome, and genetics on allergy and asthma.

The Finnish-Russian disparity in allergy prevalence was astonishing. In 2003, of the Finnish and Russian schoolchildren aged 7–16 years, 26.6% vs. 2% had positive skin prick tests to birch pollen and 28.8% vs. 4.8% to timothy grass (27). In the sensitized Russians, the allergen specific IgE levels were also lower and they showed no reactivity against 3 out of 8 timothy allergen components that were found in Finnish children (28).

For physician-diagnosed asthma, the figures were 8.8% vs. 1.6%, and for hay fever 15.6% vs. 1.2%. Food allergy was rare and peanut allergy unknown among Russian children. The disparity between the two areas had grown within time as they were somewhat larger in children than in their mothers. On both sides, parental farming and having pets in early life conferred protection against atopy as has been shown in several other studies (29).

In adults, results of the two surveys in 1998 and 2007 were also intriguing. In 1998, 5.6% of the Finns and 1.5% of the Russians reported physician-diagnosed asthma (30), in 2007, the figures were 8.3% vs. 0.7% (22). Reporting hay fever increased from 22.1% to 30.8% in Finns and from 3.9% to 5.3% in Russians. The most remarkable cohort effect was, however, observed in the sensitization rate. In adults born in 1940s, there was no difference between Finns and Russians in the sensitization to birch pollen, while Finns born in the 1980s were much more often sensitized than Russians (Figure 2, right panel). Identifying the reasons for the contrasting development would open new ways for treatment and prevention.

### 3.1. House dust mite as a surprise

Surprisingly, there was no difference in skin prick test positivity against house dust mite (*Dermatophagoides pteronyssinus*) between the Finnish and the Russian children. The latter were, however, much more frequently monosensitized, i.e. only to mite (5.2% vs. 1.4%) and without allergy symptoms while the Finns were polysensitized and symptomatic (31). Furthermore, mites tended to be abundant in Russian home dust (mean 125 mites/g dust), while being virtually absent in Finnish homes.

Mites are *ectodermal parasites* and the tropical mite (*Blomia tropicalis*), prevalent in houses of South America and Southeast Asia, even intrudes the skin. On the Russian side, the single IgE response to house dust mite, in those with low allergy prevalence, was probably a sign of a natural immune response in a mite-rich housing environment. Similar findings have been observed in rural and urban Ethiopia (32). Much of the biological role of IgE is to protect from parasite invasion and against various toxins (33). Although the relationship between parasites and allergy is still unclear, it was interesting, though not proving any causal relationship, that infection by *Ascaris lumbricoides*, the most common parasitic worm in humans, was associated with enhanced IgE responsiveness to common allergens in Russian children (34). This could be a triggering effect.

Overall, exposure to house dust mites did not explain any of the Finnish vs. Russian allergy disparity and was not causally related to the asthma and allergy epidemic (35).

### 3.2. Clues to the allergy disparities

Air pollution did not explain the Finnish/Russian allergy contrast. According to the WHO World Database in 2016, the amount of small particulate matter in the ambient air was lowest in Finland (36). Common environmental chemicals did not give an explanation either (37). Maybe the contrast was not the result of new risk factors but the consequence of losing protective factors along with the post-war *Great Accleration* (38). If so, what are the protective factors?

Originally, the *hygiene hypothesis* postulated that allergic diseases may be prevented by viral respiratory infections in early childhood (39). In adults, we measured antibodies against seven pathogens including *hepatitis A* virus, *Helicobacter pylori*, *Toxoplasma gondii*, *herpes simplex* virus, *Chlamydia pneumoniae* and the periodontal pathogens *Porphyromonas gingivalis* and *Actinobacillus actinomycetemcomitans*. Indeed, seropositivity particularly to *H. pylori* and *herpes simplex* virus (40, 41), could partly explain the difference in allergy prevalence between Finland and Russia.

**Figure 3:**
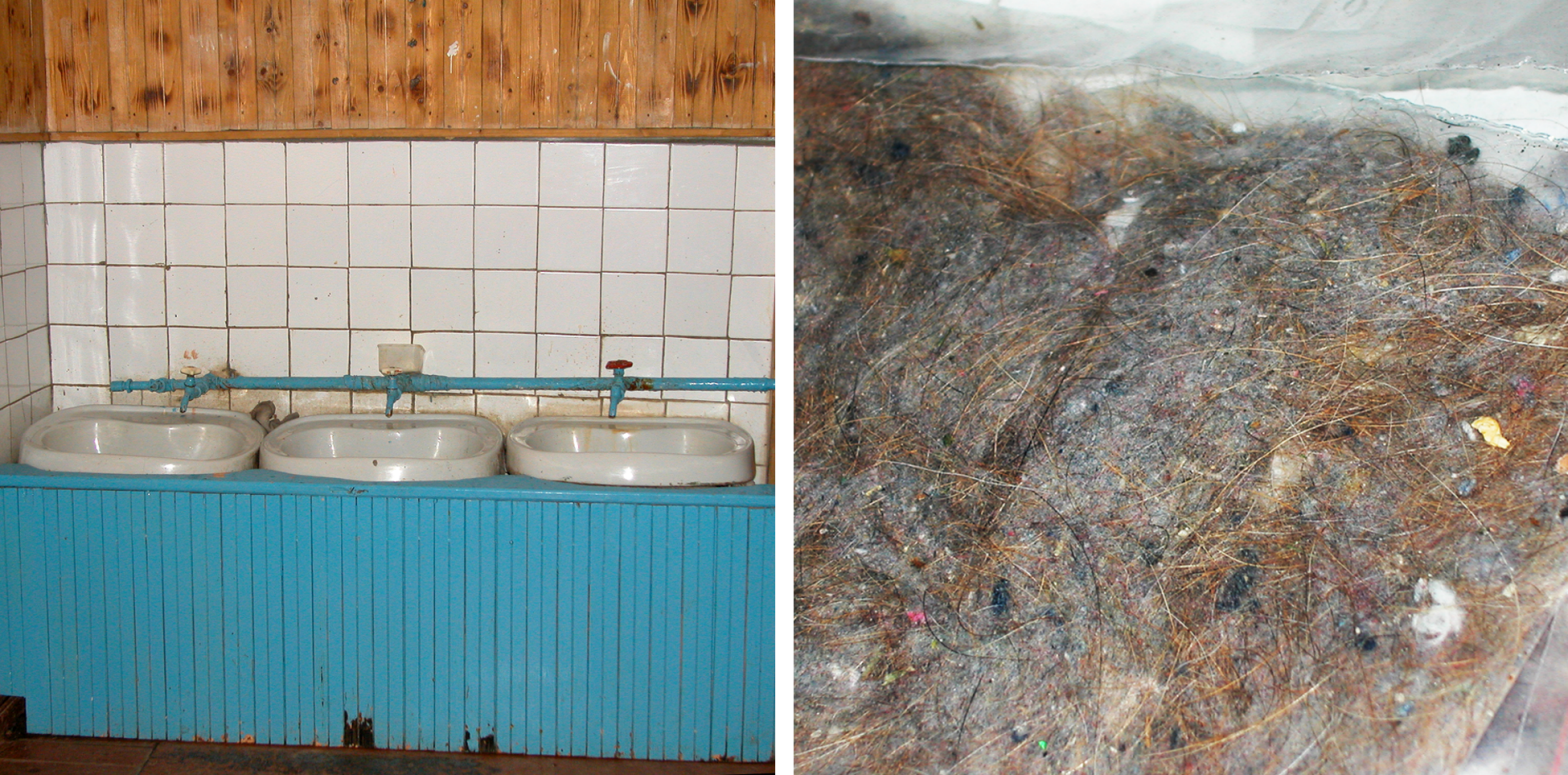
Drinking water in schools and house dust at homes contained much more microbes in Russian vs. Finnish Karelia and provided protection against allergy in Russia (42, 43). Photos: Tuula Petäys.

Did the result, however, rather indicate an overall microbial burden than any microbe-specific impact? Drinking water in Russian schools in Pitkäranta was much richer and more diverse regarding micro-organisms compared with the Finnish schools. The water in Pitkäranta schools was surface water from the lake Ladoga, not always chlorinated (Figure 3). The microbe-rich water had an independent allergy protective effect when confounding determinants were taken into account in the logistic regression analyses (42). The result was the same regarding house dust, which contained much more microbes on the Russian side compared with Finnish side (43).

### 3.3. Disconnection of man and the soil

In 2006, we discussed the role of environmental saprophytes and gut commensals, which might be major players in the immunological homeostasis (44). Graham A.W. Rook had called them *old friends* (45). Anecdotally, we also searched the data on asphalt use from 1960 to 1990 in Finland, as an indicator of built environment, and found a close correlation between asphalt use and the occurrence of asthma among Finnish young men (military conscripts). Furthermore, rapid decline in the number of farmers was associated with growing prevalence of allergic rhinitis. Both indicators reflect rapid urbanization, heavy changes in construction, land use, agriculture, forest management, and societal structures linked to them.

### 3.4. Biodiverse environment, microbiota, and allergy

In 2009, interaction between human health and the environment was addressed in a commentary article of the *butterfly theory*, the showy insects being sensitive indicators of environmental changes (46). Although exact comparative data were missing, butterflies had a wider distribution on the Russian side while an isolation and island effect, i.e., habitat fragmentation threatened the Finnish fauna (47). It seemed that in areas with high butterfly numbers and diversity, allergies were rare. In such areas, humans are more closely connected with natural environment supporting immunocompetence. The conclusion was that preserving biodiversity might have a protective effect on allergy and other diseases resulting from modern civilization. At that time, the concept of biodiversity was not really applied to public health.

A field guide for Butterflies of Britain and Europe was completed in 2011 (48) and Ilkka Hanski, ecologist and father of the so called metapopulation theory (49) provided the foreword. He joined the Karelia study group to study biodiversity in relation to allergy and asthma.

In the Finnish Karelia, the environmental biodiversity around the homes of adolescents, aged 14–18 years, was estimated by calculating the abundance of vascular plants in the yard and by characterizing the land use within a radius of three kilometer using the CORINE2000 land cover database (2). The data were correlated with skin microbiota, expression of an anti-inflammatory cytokine, IL-10, and the atopic status of the adolescents.

The results were straight forward: the higher the environmental biodiversity the richer the skin microbiota and the less atopic manifestations. A biodiverse environment is also a surrogate marker of housing and lifestyle, e.g., use of food substances in the diet, which was not specified. Nevertheless, the study showed, including only 118 subjects, strong correlations between the home environment, the microbiome, immune responses, and clinical status. The importance of green environment was further confirmed in two other cohorts of children, aged 3 and 6 years, from Finland and Estonia (50).

Especially, abundance of *gammaproteabacteria*, and on the species level, *Acinetobacter,* on the skin of the adolescents associated with allergy protection (51). The study also employed a mouse model, where intradermally applied *Acinetobacter lwoffi* induced anti-inflammatory and TH1- type gene expression and protected against allergen induced lung inflammation and increase of specific IgE.

### 3.5. Gene, microbiota, and innate immunity

The 7–11 year old Finnish and Russian children, originally surveyed in 2003, were re-examined in 2010–2012 at the age of 16–20 years (52). Allergic symptoms and allergen sensitization were still 3–10-fold more common in the Finnish subjects. The differences in allergic phenotypes, developed in early life, had remained between the two populations. In the Russian subjects, occurrence of hay fever and food allergy had remained low. Skin and nasal microbiota were highly contrasting between the populations. Especially, the genus *Acinetobacter* was abundant and diverse in Russia.

The allergy gap also called for genetic analyses. The disparity of innate immunity-related gene effects on asthma and allergy implied that living in contrasting environments associates with a different genetic profile (53). Surprisingly, in adult women (mothers of the schoolchildren), the risk allele for atopic phenotype in Finland ‒ in terms of CD14 and CC16 single nucleotide polymorphism ‒ was a protective allele in Russia (54). The opposite gene by environment interaction further emphasized the decisive role of environmental exposure and lifestyle on the allergy risk. Furthermore, the maternal genetic variants in IL-4/IL-13 pathways influenced IgE levels in the schoolchildren independently of the childrens´ own genetic effects (55). Obviously, these maternal effects were interacting with the contrasting environment and lifestyle, thereby influencing on the IgE producing capacity of the children.

Importantly, the transcriptomics analyses did not reveal any essential differences at the gene expression level that would explain the allergy disparity (56). Not unexpectedly, the function of the 261 differentially expressed genes of blood mononuclear cells (PBMC) was closely related to innate immunity, which was suppressed in the Russians compared to Finns. Moreover, long non-coding RNAs were expressed at significantly higher levels in the Russian subjects, indicating more robust gene regulation. Again, high *Acinetobacter* abundance on the skin seemed to play an important regulative role. In another comparison, the allergy gap between Finnish and Estonian children was best explained by disparate early exposure to environmental microbes, especially to the genus *Acinetobacter* (57).

Overall, the Russians had richer gene-microbe networks and interaction than the Finns, which could be linked to a more balanced innate immunity and related with lower allergy prevalence.

### 3.6. Epigenetic adaptation

The genotype profiles are like computer “hardware”, preserving the individual information for life. Epigenetic programming, the “software”, regulates to what extent this information is open to translating genes to function (i.e., gene transcription followed by protein synthesis). Allergic disease is associated with epigenetics, i.e., chemical DNA modifications that regulate gene expression — and these modifications are induced by environmental exposures. Thus, epigenetics serves as environmental biomarkers linking our genes with exposure and disease. The epigenetic reading of the *exposome* (58) modulates gene-environment interaction and have a central role in immune homeostasis.

Methylation is one of the main epigenetic mechanisms. *CD14 methylation*, responding to endotoxins as markers of environmental microbial load, differed significantly between the Finnish and Russian subjects (59).

In the PBMCs, there were also marked differences in the expression of so called long non-coding RNAs (lncRNA, not coding protein synthesis) (60). The Russian subjects showed upregulation of 37 lncRNAs, which are part of the co-expression network with 20 genes known to be related to allergic disease. All these genes are also components of pathways corresponding to cellular response to bacteria. The upregulation of the lncRNAs was positively correlated with abundance of *Acinetobacter* and also associated with innate immune suppression in the Russian subjects, who showed less likely harmful response to common allergens. Thus, the function of lncRNAs seems essential in immune-microbiota crosstalk, although it is not fully understood.

In the birch-pollen-allergic individuals, the activated innate immunity networks during *in vitro* allergen stimulation mimicked those activated during viral infections (61). It looked like the immune system of the allergic subjects misread the birch-pollen proteins as potential viruses.

### 3.7. Examples of other population contrasts

Many other comparisons exploring allergy disparities among populations have been carried out in Europe and elsewhere. In Karelia, another Finnish-Russian Study Group made surveys for type 1 diabetes and found it sixfold more common on the Finnish side (62). They also looked for allergy among schoolchildren and found less sensitization and more microbial load on the Russian side (63).

Already in 1992, the lower prevalence of allergic disorders in former Eastern Germany, Leipzig, compared with Munich, was speculated to associate with Western lifestyle and living conditions (64). This was in accordance with the observations from children in Eastern Europe having less atopy-related disorders than those living in Scandinavia (65). Especially, the composition of intestinal microflora during the first year of life seemed to modify the allergy risk (66).

A population-based study in Mongolia showed that rural lifestyle protected from allergic rhinitis and sensitization (67). In US, the traditional farming practices protected the Amish children from asthma by shaping their microbial load and innate immune response, compared with the Hutterites with more industrialized type of agriculture (68).

## 4. From hypothesis to practice

### 4.1. The Finnish Allergy Programme 2008–2018

Does the biodiversity hypothesis work in practice? The burden of allergic disease and asthma has been growing for decades in Finland and elsewhere, which implies that prevention strategies at the population level have failed. The Finnish 10-year, nationwide action plan to mitigate the allergy burden was based on clinical experience and the Karelia study results (69, 70). It was also a continuum of the Asthma Programme 1994–2004 (17).

The real-world intervention aimed to improve immunological tolerance, encourage nature contacts, and promote allergy health, i.e., wellbeing even with allergic disease (Table 1). Jean Bousquet wrote: “In allergy, a new day has begun” (71). A major effort was taken to educate healthcare workers and to inform patients, families, and lay-public.

**Table 1:** The Finnish allergy programme 2008–2018. Practical advice to prevent allergic disease and asthma (69, modified).

Primary prevention	Secondary, tertiary prevention
• Support *breastfeeding*, introduce solid foods from 4 to 6 months. • Do not avoid environmental exposure unnecessarily (e.g., foods, pets). • *Educate immunity by diverse connection to natural environment* (eating, drinking, breathing, touching). • Strengthen immunity through a *healthy diet* (e.g., traditional Mediterranean or Baltic type). • Strengthen immunity by regular *physical exercise*. • Use *antibiotics* with care. Most microbes are useful and support health. • Beware of *probiotic bacteria* in fermented food or other preparations, they may strengthen immunity. • Do not smoke, avoid tobacco smoke.	• *Targeted avoidance*. Avoid allergens only, if diagnosed and considered a significant risk. • *Strengthen immunity by increasing exposure to natural environment*. • A healthy diet (Mediterranean or Baltic type) improves allergy and asthma control. • Regular physical exercise improves asthma control. • Consider use of fermented food or other preparations including probiotic bacteria. • Consider *allergen specific immunotherapy*: allergens as they are (foods), sublingual tablets or drops (e.g., pollen, house dust mite), subcutaneous injections. • Control respiratory and skin *inflammation* early and effectively. Make sure *long-term control*. • Consider *biologicals* in severe cases. • Stop smoking and tobacco smoke.

The Programme reached most of its quantitative goals, e.g., food allergy diets, work-related allergies and asthma hospital days halved (72, 73). Prevalence of asthma, rhinitis and eczema levelled off (74, 75). In 10 years, about €2 million were invested in education and public information, not including the major voluntary work by healthcare professionals. The cumulative savings were €1.2 billion in direct healthcare and indirect disability costs (76).

### 4.2. Testing the biodiversity hypothesis

The Finnish Allergy Programme was an open, non-controlled public health intervention including all citizens and employing real-world data and questionnaire surveys to evaluate outcomes. We cannot be sure whether the favourable result would have emerged even without the Programme. Thus, controlled studies to show benefits of improved nature relatedness are necessary.

In a controlled intervention study, daycare yards were enriched by soil blocks and other natural elements (77). In four weeks, the skin microbiota of the intervention group was enriched with immunomodulatory effects. Importantly, continuing the biodiversity intervention up to two years sustained the enriched microbiota in children (78).

The daycare study was continued with a placebo-controlled, double-blinded intervention where the playground sand was enriched with microbially diverse soil (79). Visually similar, but microbially poor sand was used in the placebo group. At two and four weeks, skin bacterial richness and diversity were higher in the intervention than placebo group with immunomodulatory effects. The result supported the biodiversity hypothesis of immune-mediated diseases.

### 4.3. Nature step to health – Lahti regional health and environment programme 2022–2032

**Figure 4:**
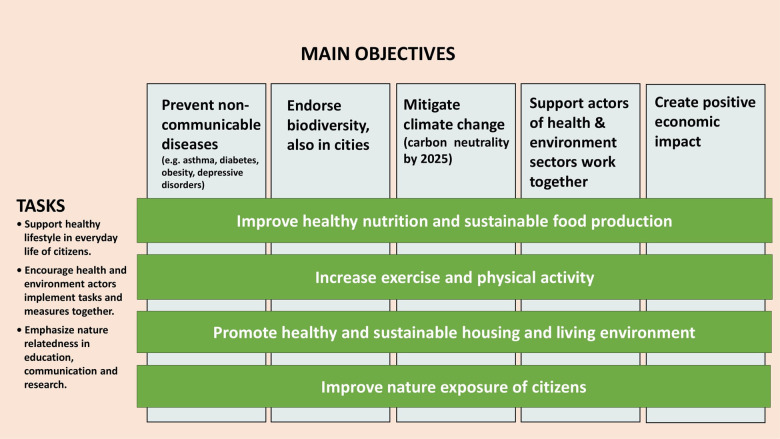
Nature step to health — Lahti regional health and environment programme 2022–2032. The action plan combines disease prevention and actions for environmental sustainability (80).

The City of Lahti in Southern Finland with 120 000 inhabitants was nominated the EU Green Capital 2021 for its long-term work for sustainable environment. *Nature Step to Health* is a 10-year Health and Environment Programme in the Lahti region to combine public health promotion with stopping nature loss and mitigating climate change (Figure 4) (80, 81).

The non-communicable diseases chosen as specific indicators for the Lahti Programme are *asthma, diabetes, obesity, and depression*, but data for cardiovascular and autoimmune diseases are also followed. The asthma preventive effect of farming environment has been convincingly shown (82). Altered microbiome precedes or associates with types 1 and 2 diabetes (83, 84), cardiovascular disease (85) or autoimmune diseases (86). The risk of type 1 diabetes is decreased in children with disease associated HL-DQ alleles, who are exposed to an agricultural environment early in life (87). A protective effect of residential greenness on major depressive disorder (88) and obesity (89) has come up in observational studies. Recently, the risk of early-onset cancers (adults <50 years) has been linked to early-life environmental exposures and microbiome (90).

The practical actions concern all citizens to favor healthy diet, increase physical activity and mobility, improve housing environment, and encourage nature contacts. These actions are also prescriptions for planetary health (7, 91). A new kind of educational effort for healthcare and information for lay public is taking place. The Päijät-Häme Joint Authority for Health and Wellbeing implements the Programme together with the City of Lahti and Lahti University Campus. Real-world data are employed from official registers, and questionnaire surveys are carried out. Cross-sectional research, new services and *nature-based solutions* for environmental sustainability are promoted.

## 5. Conclusions – from great acceleration to great slowdown

Factors influencing on immune regulatory circuits originate from the evolutionary home of humankind: soil, natural waters, and ambient air. Understanding the interaction of the ecosystem of human body with the surrounding ecosystems, especially with microorganisms and biogenic compounds, would give clues both for primary and secondary prevention of the diseases of affluence.

The increase of non-communicable diseases (NCDs), e.g., *asthma, allergic disease, diabetes, obesity, depressive disorders, early cancer, even Alzheimer disease* (92) has occurred in parallel with urbanization and changes of environment and lifestyle. In the Finnish and Russian Karelia, transcriptome studies did not explain the allergy and asthma disparity while microbiome changes explained much of these epidemiologic trends. The main reasons seemed to be a contrasting exposure to microbiota in the living environment, especially in early life, and differences in adaptive epigenetic regulation. In urban environments, the microbial communities of children and even pets tend to be poor, as compared with rural individuals (93–95). Already in 2009, it was shown that urban house dust elicits a Th2-type response whereas barn dust with high bacterial diversity directs the cells towards a Th1-type response (96).

The biodiversity hypothesis was preceded by the hypotheses of hygiene (39), old friends (45), microbial diversity (97), and microbial deprivation (98). As biodiversity concerns both the macro- and microworld, the extreme complexity is obvious (99). In epidemiological studies, biodiversity has not, until recently, been determined either as a main or confounding variable. It has been difficult to agree upon any satisfactory *biodiversity index* to describe the natural richness and variety of the living environment (100). Nevertheless, CORINE land cover database by the European Environment Agency gives an idea of natural richness of our surroundings. It is standardized, available free of charge, and updated in 2018 (101).

Is the biodiversity hypothesis relevant for actions stopping the allergy & asthma epidemic or endorsing public health in general? The only long-term implementation so far is the Finnish Allergy Programme 2008–2018, which aimed to improve immune tolerance and nature relatedness. The nationwide intervention was not performed in a controlled setting, but disease burden was significantly mitigated with major cost savings. In 2022, the Programme was acknowledged by the EU commission in the *Best Practice Portal* of non-communicable diseases (NCDs).

Although biodiversity interventions are at an early stage (102), promising results from controlled studies have been published (79). Human microbiome and immune balance can be modified in a relatively short period of time, in weeks and months, but it remains to be shown whether interventions truly prevent symptoms or clinical disease. Nonetheless, there is enough evidence to recommend safe contacts with biologically rich environment for children. They are also important for elderly as populations are aging rapidly in Europe, China (103) and elsewhere. Recent data from Belgium indicated that urban green spaces may even protect against suicide mortality (104).

In the medical treatment of non-communicable diseases, enhancing immune tolerance and preventing or blocking inflammatory pathways are increasingly favoured. In allergy, oral immunotherapy (OIT) against pollen and mite is common practice and increasingly important in food allergy (105). Allergen components diagnostics is part of good practice and has improved targeted allergen avoidance and prevention in general (82). In severe allergy and asthma, biologicals are already a common place. Even in cancer treatment, an immunological approach using mRNA vaccines is breaking through (106).

Back to the tuberculosis sanatoria! Before the curative anti-tuberculosis medication, treatment improved hygiene conditions, provided nutritious food, fresh air (aerotherapy), sunlight (heliotherapy), and a mixture of bed rest, physical activity and work to strengthen the immune system (107). The sanatoria were built in beautiful, naturally rich environments (in Finland, 17 sanatoria in 1930s). Now we know that the natural air provides more than 1,000 biogenic chemicals (volatile organic compounds), which may have anti-inflammatory, anti-oxidative and anxiolytix effects (108, 109). However, true immunological and clinically meaningful effects remain to be shown.

In conclusion, the increase of asthma, allergic diseases and other NCDs is mainly caused by changes in environment and lifestyle. They can be influenced by a healthy diet, physical exercise, preferably in wider nature, green housing environment, and nature contacts. We are protected by two nested layers of biodiversity, the inner layer consisting of microorganisms residing our bodies and the outer layer of living surroundings (110). We are not going “back to nature”, but we can modify and enrich our lifestyle and environment with natural elements. Urban environments should be supplied with variable nature-based solutions to endorse green infrastructure. The biodiversity in cities can be partly restored by favouring and expanding green areas with trees and forest-like environment (111).

**Figure 5:**
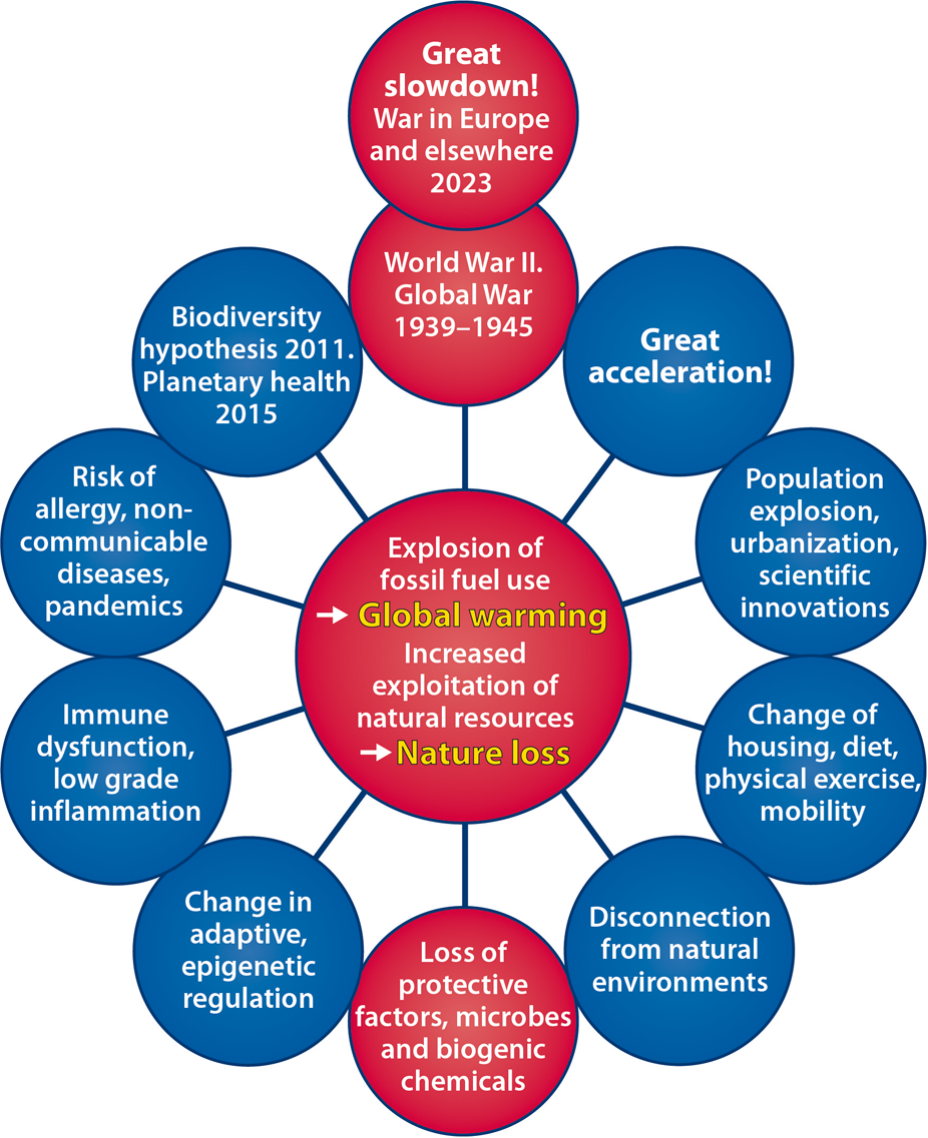
Health wheel going around. From the disaster of World War II to **great acceleration** with population explosion, urbanization, and changes of environment and lifestyle. Overuse of natural resources has kept the wheel going but is causing climate crisis and nature loss with major health impact. Coming around to ongoing military conflicts in Europe and elsewhere denotes **great slowdown**.

The Health Wheel after the World War II is outlined in Figure 5: from global war destruction to *great acceleration*, population explosion, urbanization, and overuse of natural resources to *great slowdown*. The COVID-19 pandemic showed the priorities of the societies: health and safety come first. For a more sustainable future, *integrated prevention* of human disease, climate crisis, and nature loss with the new technical and scientific innovations makes sense (112, 113). Management of the environmentally-driven allergic and respiratory disease may demonstrate the transition and pave the way guided by the Helsinki Alert 2015 (114) and Declaration 2020 (115).

## On behalf of

Matti Ahlström, Harri Alenius, Maria A. Andersson, Petri Auvinen, Angelica Berger, Pierre Candelaria, David Chandler, Jukka Corander, Vittorio Fortino, Nanna Fyhrquist, Jack Goldblatt, Dario Greco, Tari Haahtela, Ilkka Hanski†, Catherine M. Hayden, Ilkka Helenius, Leena von Hertzen, Anne Hyvärinen, Hanna Jarva, Sarra E. Jamieson, Pekka Jousilahti, Piia Karisola, Antti Karkman, Anne M. Karvonen, Siew-Kim Khoo, Hannu Kiviranta, Mikael Knip, Jyri-Pekka Koskinen, Kaisa Koskinen, Timo U. Kosunen†, Tiina Laatikainen, Vilma Lahti, Sirpa Laitinen, Khui Hung Lee, Jenni Lehtimäki, Sari Lehtimäki, Maili Lehto, Joona Lehtomäki, Marina Leino, Peter N. LeSouef, Marja-Leena Majuri, Pekka Malmberg, Olga Markelova, Vladimir Masyuk, Tuula Metso, Robert Movérare, Mika J. Mäkelä, Joseph Ndika, Aino Nevalainen, Jari Niemelä†, Onni Niemelä, Noora Ottman, Laura Paalanen, Jaakko Pakarinen, Vladimir Pantelejev, Lars Paulin, Pirkka T. Pekkarinen, Sirpa Pennanen, Tuula Petäys, Juha Pekkanen, Tarja Pitkänen, Pirkko J. Pussinen, Lasse Ruokolainen, Paula Rytilä, Mirja Salkinoja-Salonen, Ossian Saris, Terhi Savinko, Giovanni Scala, En Nee Schultz, Kristiina Sirola, Yong Song, Alina Suomalainen, Vallo Tillman, Kaisa Torppa, Mihail Uhanov, Erkki Vartiainen, Ville Veckman, Johanna Vendelin, Tiina Vlasoff, Lukas Wisgrill, Henrik Wolff, Guicheng Zhang, Elvira K. Zilber.

## Author contributions

All authors have participated in the Karelia Allergy Study, the Finnish Allergy Programme or the other Finnish Public Health Interventions. They have actively contributed to the writing process or commented the paper. All authors contributed to the article and approved the submitted version.

## Conflict of interest

The authors declare that the research was conducted in the absence of any commercial or financial relationships that could be construed as a potential conflict of interest.
